# Illustrations of Osteology

**Published:** 1839-04

**Authors:** 


Art. XII.—*Illustrations of Osteology.* By Theodore S. G. Boisragon, m.d.—*London,* 1839. Large folio.

This work consists of plates beautifully drawn and lithographed by Dr. Theodore Boisragon, with corresponding sketches in outline, in which all the individual parts are named and briefly described. The present fasciculus contains three plates; and we believe that two more plates, to be published shortly, will complete the small work. Many years since, when the actual subjects of anatomical study were less generally accessible, these plates would have been invaluable: they will still be found very useful to the solitary student, who is making preparation for his career at some great school. Dr. Boisragon shows, in these plates, such excellent skill and taste, that we regret his talents were not employed on a more important and more promising undertaking.

